# Supportive Care and Unmet Needs in Upper Gastrointestinal Cancer Patients: Screening and Related Factors

**DOI:** 10.3390/ijerph18158124

**Published:** 2021-07-31

**Authors:** Ya-Ting Tseng, Chia-Hsun Hsieh, Chien-Wei Hung, Chia-Chia Chen, Shu-Hui Lee, Li-Yun Lee, Shu-Ching Chen

**Affiliations:** 1 Department of Nursing, Chang Gung Memorial Hospital at Linkou, Taoyuan 333, Taiwan; alu660109@cgmh.org.tw (Y.-T.T.); nweix@cgmh.org.tw (C.-W.H.); jajad@cgmh.org.tw (C.-C.C.); f22028@cgmh.org.tw (S.-H.L.); 2 Division of Hematology/Oncology, Department of Internal Medicine, Chang Gung Memorial Hospital at Linkou, Taoyuan 333, Taiwan; wisdom5000@gmail.com; 3 Division of Hematology/Oncology, Department of Internal Medicine, New Taipei Municipal Tucheng Chang Gung Memorial Hospital, New Taipei 236, Taiwan; 4 Department of Medicine, College of Medicine, Chang Gung University, Taoyuan 333, Taiwan; 5 Department of Nursing, College of Nursing and Health Sciences, Da-Yeh University, Changhua 515, Taiwan; leeliyun@mail.dyu.edu.tw; 6 School of Nursing and Geriatric and Long-Term Care Research Center, College of Nursing, Chang Gung University of Science and Technology, Taoyuan 333, Taiwan; 7 School of Nursing, College of Medicine, Chang Gung University, Taoyuan 333, Taiwan; 8 Department of Radiation Oncology and Proton and Radiation Therapy Center, Chang Gung Memorial Hospital at Linkou, Taoyuan 333, Taiwan

**Keywords:** upper gastrointestinal cancer, screening, supportive care needs, unmet needs, social support

## Abstract

Upper gastrointestinal (UGI) cancer treatment can cause physical and psychological distress and may result in unmet needs. The purposes of this study were to (1) examine the levels of gastrointestinal (GI) symptom distress, social support, and supportive care needs; (2) screen the priorities of unmet supportive care needs; and (3) identify the factors associated with supportive care needs among UGI cancer patients receiving chemotherapy. This cross-sectional study examined UGI cancer patients who received treatment from the outpatient chemotherapy department of a single cancer center in northern Taiwan. Questionnaires were used to collect data regarding GI symptom distress, social support, unmet needs, and supportive care needs. The top three unmet needs were “fears about the cancer spreading”, “uncertainty about the future”, and “being informed about things you can do to help yourself to get well”. Descriptive statistics examined the levels of GI symptom distress, social support, supportive care needs, and priorities of unmet supportive care needs. Stepwise regression was conducted to determine significant factors related to supportive care needs. Greater supportive care needs were found to be associated with higher levels of disease-related worries, increased treatment-related symptoms, and a lower level of physical performance. These factors explained 48.0% of the variance in supportive care needs. Disease-related worries and treatment-related symptoms strongly influence overall supportive care needs and each domain of supportive care needs. Symptom management and psychological support for patients receiving outpatient chemotherapy may help patients meet needs.

## 1. Introduction

Cancer is a leading cause of mortality and morbidity worldwide. Gastric cancer, pancreatic cancer, and cholangiocarcinoma are the most common cancers of the UGI tract and accessory organs of digestion [1]. There are a growing number of new cases worldwide. There is significant geographic variation, and more than half of the new cases are occurring in Eastern Asia, including Taiwan [2,3]. Gastric cancer, pancreatic cancer, and cholangiocarcinoma are also most common UGI cancer and cause of mortality in Taiwan [4,5,6]. Surgery or surgery combined with chemotherapy (CT) are the major treatments for UGI cancer [7]. UGI and its treatment causes varying levels of symptom distress [7], social function decline [8], and disease-related worry [9], which may result in unmet supportive care needs [10].

Supportive care needs refer to patients’ perceived needs for help and support in multifaceted domains, including psychological, health information, physical and daily living, patient care and support, and sexuality needs [11,12]. Research has examined unmet supportive care across various cancer populations [13]. Information needs were highest at cancer diagnosis, while physical and daily living needs were highest during treatment [14]. Unmet needs are associated with younger age [15], advanced cancer stage [16], more symptom distress [15], higher levels of anxiety [15,17] and depression [15], better physical functioning [15], and being single or widowed [17]. Although previous studies have explored these issues, most research has focused on unmet needs [14,15]; hepatocellular carcinoma patients [15], gastric cancer [9,14], and head and neck cancer [17,18] who undergoing treatment, and those who above-mentioned study subjects who were hospitalized. Despite the fact that previous studies have explored these issues, most research has focused on patients in treatment who had gastric cancer [14], hepatocellular carcinoma [15], and oral cancer [17,18] in Taiwan. There has been a lack of study exploring the symptom distress, social support, unmet needs, and supportive care needs among gastric cancer, pancreatic cancer, and cholangiocarcinoma patients. UGI patients receiving outpatient chemotherapy may experience insufficient service or support to deal with particular health problems. The need for such care reflects a feeling experienced when subjective perceptions are inconsistent with a desired state [19]. Screening for, and identification of, unmet supportive needs in an outpatient unit is an important issue in cancer care. Insufficient attention has been devoted to this important topic. On the basis of the literature review, we hypothesized that these patients may also experience symptom distress [15], psychological distress [15,16], and insufficient of social support [17], resulting in unmet needs while receiving outpatient chemotherapy among gastric cancer, pancreatic cancer, and cholangiocarcinoma patients, especially younger patients [15] and those who had advanced cancer stage [16]. Therefore, the purposes of this study were to (1) examine the levels of gastrointestinal (GI) symptom distress, social support, and supportive care needs; (2) screen the priorities of unmet supportive care needs; and (3) identify the factors associated with supportive care needs among UGI cancer patients receiving chemotherapy.

## 2. Materials and Methods

### 2.1. Design and Population

#### 2.1.1. Design

This study used a cross-sectional, descriptive, and correlational design and followed the STROBE checklist. Consecutive sampling was conducted to recruit subjects from the outpatient chemotherapy unit of a medical center in northern Taiwan, from February 2019 through July 2019.

#### 2.1.2. Study Subjects

The inclusion criteria were (1) new diagnosis of UGI cancer (gastric cancer, pancreatic cancer, or cholangiocarcinoma); (2) receiving at least two cycles of initial outpatient chemotherapy within the last three months; (3) agreeing to participate in the study after receiving an explanation of its purposes and procedures; and (4) were 20 years of age, or older. Patients with mental disorders, an unstable systemic disease (e.g., active infection or other underlying disease), recurrent UGI cancer, or physical performance less than 60 on the Karnofsky Performance Status (KPS) scale [20] were excluded. Participants received outpatient chemotherapy for 4–6 months, with fluoropyrimidine-based other gemcitabine-based regimens biweekly, triweekly, or monthly [7,21,22].

### 2.2. Ethical Considerations

Approval from the Institutional Review Board of the medical center (number: 103-4464B) and a permission certificate were obtained. All participants signed informed consent forms prior to data collection.

### 2.3. Data Collection

Data was collected directly from patients receiving chemotherapy at the outpatient chemotherapy unit. Potential subjects and all those who agreed to participate provided written consent and were given a detailed explanation of the research purpose and procedures. A research nurse assisted the patients with completing the questionnaires at their bedside in the chemotherapy room, which required approximately 10–15 min per patient.

### 2.4. Measures

#### 2.4.1. Supportive Care Needs Survey-Screening Tool

The Supportive Care Needs Screening Tool (SCNS-ST9) is an ultra-short screening instrument consisting of nine questions derived from the Supportive Care Needs Survey Short Form (SCNS-SF34) by Girgis et al. [12]. The SCNS-ST9 was used to screen unmet supportive care needs of patients throughout their chemotherapy. The SCNS-ST9 consists of 9 items screening 5 subscales: psychological needs (2 items), health information needs (2 items), physical and daily living needs (2 items), patient care and supportive needs (2 items), and sexuality needs (1 item). The respondents rate their needs from 1 (no need) to 5 (high need for help). The summed scores of each domain, and the global scale, are converted into standardized scores ranging from 0 to 100, with higher scores representing more supportive care needs. Individual items for “no need”, “needs already satisfied”, and “some need” indicated that the need was met, whereas individual items for “low needs” and “moderate or high needs” indicated unmet needs [12]. The SCNS-ST9 was translated into Chinese. Hu et al. [23] demonstrated that the SCNS-ST9 has adequate psychometric properties in a Chinese cancer population. Cronbach’s alpha was 0.88 for this study.

#### 2.4.2. Social Support Scale

Social support was measured by the Medical Outcomes Study social support survey (SSS) [24]. The 19-item SSS consists of four subscales: emotional/informational support (8 items), tangible support (4 items), affectionate support (3 items), and positive social interaction (3 items), with 1 additional item. The score for each item ranges from 1 (not at all) to 5 (very much). The summed scores of each domain and the global scale are converted into standardized scores ranging from 0 to 100, with higher scores representing greater support. Several studies in Taiwan reported satisfactory psychometric properties [25,26,27]. Cronbach’s alpha coefficient of the study data was 0.95.

#### 2.4.3. European Organization for Research and Treatment of Cancer—Gastrointestinal-Related Neuroendocrine Tumors

GI-related symptom distress was measured by the EORTC-QLQ GINET21. The EORTC-QLQ-GINET21 contains a total of 21 items: four single-item assessments relating to muscle/bone pain (MBP), body image (BI), information/communication function (INF), and sexual functioning (SX), together with 17 items organized into five proposed scales: endocrine symptoms (ED; three items), GI symptoms (GI; five items), treatment-related symptoms (TR; three items), social functioning (SF) of the new module (SF21; three items), and disease-related worries (DRW; three items) [28]. Scores were recorded as 0 to 100, using the EORTC guidelines, with higher scores reflecting more severe symptom distress or more problems. Yadegarfar et al. [29] showed that Cronbach’s α coefficient was >0.7 for all parts of the QLQ-GINET21 at 6 months. Intraclass correlation was >0.85 for all scales. Discriminant validity was confirmed, with values < 0.70 for all scales compared with each other. The EORTC-QLQ GINET21 has been translated into several languages and has reported satisfactory psychometric properties [30]. Cronbach’s alpha for the EORTC-QLQ-GINET21 for this study was 0.93.

#### 2.4.4. Karnofsky Performance Status Scale

Physical performance status was assessed using the Karnofsky Performance Status (KPS) scale, which ranges from 100% (normal function) to 0% (death) [20]. The KPS has been widely used in studies of cancer patients in Taiwan [31,32].

#### 2.4.5. Demographic and Clinical Characteristics Form

The demographic data included age, gender, employment after diagnosis, marital status, educational level, religion, smoking, and drinking. Clinical characteristics included cancer diagnosis, cancer stage, medical treatment, and time since cancer diagnosis.

### 2.5. Statistical Analysis

SPSS version 26.0 (IBM Corp., Armonk, NY, USA) was used for data analysis. Demographic and clinical characteristics, levels of GI symptom distress, social support, supportive care needs, and the prevalence of unmet supportive care needs were summarized by descriptive statistics (number and percentage, means, median, and interquartile range (IQR)). Items of SCNS-ST9 scored 1–3 indicated that the need was met; those scored 4–5 indicated unmet needs [12]. Normality of all data was tested before conducting inferential statistics. Parametric testing was used for inferential statistics. Stepwise linear regression analysis was conducted to identify factors related to supportive care needs and five specific aspects of supportive care needs. The independent variables were as follows: age, marital status (without partner vs. with partner); religion (no vs. yes); cancer stage (early vs. advanced); physical performance; muscle/bone pain (MBP); body image (BI); information/communication function (INF); sexual function (SX); endocrine symptoms (ED); GI symptoms (GI); treatment-related symptoms (TR); social functioning (SF); disease-related worries (DRW); and social support.

### 2.6. Sample Size

Sample size was calculated using a statistical power analysis for multiple regression [33]. The size of the statistical sample achieved 80% power to detect an *R*^2^ of 0.15 attributed to 7 independent variables with a significance level (α) of 0.05. Therefore, 107 subjects, at most, were needed to meet the criteria.

## 3. Results

### 3.1. Demographic and Clinical Characteristics

The research nurse identified eligible participants from patient registration lists of the relevant hospital/organization. Patients were selected while receiving chemotherapy, and potential eligible subjects were referred by their physician. A total of 100 upper UGI cancer patients were recruited into this study. Of 103 eligible patients approached, 3 patients dropped out or declined to participate because they had no time or interest. The response rate was 97.1%.

The average age of patients in the study was 60 (median = 62, interquartile range, IQR = 55–67) years. Demographics were as follows: male (n = 58, 58.0%); married (n = 95, 95.0%); elementary school-level education (n = 30, 30.0%); held Buddhist/Taoist religious beliefs (n = 66, 66.0%). Clinical characteristics were that gastric cancer was the most common cancer (n = 43, 43.0%). Additional clinical characteristics included stage IV disease at diagnosis (n = 53, 53.0%); newly diagnosed cancer (n = 94, 94.0%); received only chemotherapy (n = 45, 45.0%); high (90–100) KPS scores (n = 74, 74.0%) (Table 1).

### 3.2. Levels of GI Symptom Distress, Social Support, and Supportive Care Needs

The mean scores for each subscale were as follows: muscle/bone pain (MBP), 12.33 (median = 0, IQR = 0–33.33); body image (BI), 13.33 (median = 0, IQR = 0–33.33); information/communication function (INF), 50.02 (median = 66.67, IQR = 33.33–66.67); sexual function (SX), 11.33 (median = 0, IQR = 0–25); endocrine symptom (ED), 5.22 (median = 0, IQR = 0–11.11); GI symptom (GI), 12.88 (median = 13.33, IQR = 0–20); treatment-related symptom (TR), 16.27 (median = 11.11, IQR = 0–27.78); social functioning (SF), 16.11 (median = 11.11, IQR = 11.11–22.22); disease-related worries (DRW), 23.68 (median = 22.22, IQR = 11.11–22.22).

The overall mean social support score was 90.87 (median = 96.05, IQR = 87.17–100). The descending ranking for each subscale was as follows: affectionate support (mean = 92.83, median = 100, IQR = 91.67–100); positive social interaction (mean = 90.25, median = 100, IQR = 83.33–100); emotional/informational support (mean = 79.83, median = 86.11, IQR = 75.69–88.89); tangible support (mean = 40.97, median = 44.44, IQR = 38.89–44.44).

The overall mean supportive care needs score was 34.25 (median = 25, IQR = 19.44–35.42). The descending ranking for each subscale was as follows: health information needs (mean = 34.75, median = 25, IQR = 25–34.38); psychological needs (mean = 34.13, median = 25, IQR = 25–50); sexuality (mean = 29.72, median = 0, IQR = 0–25); physical and daily living needs (mean = 27.13, median = 25, IQR = 25–50); patient care and supportive needs (mean = 13.00, median = 25, IQR = 25–50) (Table 2).

### 3.3. Screening of Unmet Supportive Care Needs

The SCNS-ST9 screened the priorities of unmet supportive care needs. The degree of patients’ supportive care needs were reported as met and unmet on the SCNS-ST9. Items scored 1–3 indicated that the need was met; those scored 4–5 indicated unmet needs [8]. The top three unmet needs were “fears about the cancer spreading” (15.0%), “uncertainty about the future” (14.0%), and “being informed about things you can do to help yourself to get well” (11.0%; Table 3).

### 3.4. Factors Associated with Supportive Care Needs

Multiple regression analysis identified factors that were significantly and independently associated with supportive care needs and five aspects of supportive care needs. Patients who had a greater occurrence of disease-related worries (β = 0.428), more treatment-related symptoms (β = 0.331), and lower physical performance (β = −0.188) were more likely to have greater overall supportive care needs. These three factors explained 48.0% of the total variance in this parameter. Patients who had a higher incidence of disease-related worries (β = 0.377), more treatment-related symptoms (β = 0.251), less endocrine symptoms (β = −0.185), poor social functioning (β = 0.234), and who were younger age (β = −0.149) were more likely to have greater psychological needs. These five factors explained 53.9% of the total variance in this parameter. Patients who had poor social functioning (β = 0.362), more treatment-related symptoms (β = 0.183), and more endocrine symptoms (β = 0.191), as well as those who were married (β = 0.175) were more likely to have greater health information needs. These four factors explained 33.2% of the total variance in this parameter. Patients who had negative body image (β = 0.255), poor social functioning (β = 0.218), less physical performance (β = −0.303), and more treatment-related symptoms (β = 0.183) were more likely to have greater physical and daily living needs. These four factors explained 39.0% of the total variance in this parameter. Patients who had greater disease-related worries (β = 0.313) and more sexual function problems (β = 0.233) were more likely to have greater patient care and supportive needs. These two factors explained 20.4% of the total variance in this parameter. Patients who had greater disease-related worries (β = 0.204), more sexual function problems (β = 0.272), early cancer stage (β = −0.203), and increased treatment-related symptoms (β = 0.216) were more likely to have greater sexuality needs. These four factors explained 33.8% of the total variance in this parameter. Disease-related worries and treatment-related symptoms were the most common factors associated with overall supportive care needs and with all individual domains of supportive care needs (Table 4).

## 4. Discussion

Our study verified that patients reported “fears about the cancer spreading (psychological domain)” was the top priority of unmet supportive care needs, followed by “uncertainty about the future (psychological domain)” and “being informed about things you can do to help yourself to get well (health information domain)”. We also revealed that patients had mild-to-moderate supportive care needs, and “psychological needs” was the top supportive care needs, followed by “health information needs”. These results are inconsistent with those of a previous study [14], which reported that information needs were highest at cancer diagnosis, while physical and daily living needs were highest during treatment. In clinical care, outpatient chemotherapy information package should be developed to provide for these patients, satisfying their needs for information and helping to reduce associated psychological needs.

This study found that patients reported a high level of information/communication function, with mean = 50.02 (median = 66.67, IQR = 33.33–66.67), which is higher than a previous study [29]. Yadegarfar et al. [29] reported that pancreatic cancer patients’ information/communication function (INF score) was 10 before treatment, 4 at 3 months after treatment, and 10 at 6 months after treatment. Patient information regarding other sites of GI neuroendocrine cancer and information/communication function (INF score) was 13 before treatment, 11 at 3 months after treatment, and 10 at 6 months after treatment. This difference may be due to a difference in the cancer types and setting of various studies. Yadegarfar et al. [29] included GI neuroendocrine cancer from inpatient treatment, whereas our subjects received outpatient chemotherapy. Clinical healthcare professionals should actively evaluate patients’ information and communication concerns before and during treatment and provide available information and discussion to address patients’ information and communication problems and concerns.

Patients in our study perceived higher information needs, particularly those who received at least two cycles of initial outpatient chemotherapy within the last three months. These findings are consistent with earlier studies [14,15]. Patients had a high level of information needs, related to managing illness and side effects, during transition from hospital to home, and when their cancer was under control or diminishing. This finding may be explained by the fact that our study subjects’ schedule in the outpatient department often caused insufficient time for discussing information in one sitting, which might have impacted meeting supportive care needs. Telephone counseling and electronic learning may be useful in meeting these information needs.

Results of the present study indicated that patients who had more disease-related worries, more treatment-related symptoms, and lower physical performance were more likely to have overall supportive care needs. These findings are consistent with those of prior studies [9,10]. Patients in this study undergoing active anti-cancer therapy at the outpatient department, and experiencing side effects of chemotherapy, expressed symptom distress and showed declining physical performance. In addition, most patients were concerned with treatment efficacy of their chemotherapy regimen and worried about their cancer progressing during this period [9]. Symptom management, as well as patient education about physical activities, may help patients alleviate symptoms and promote patients’ self-perception of a more positive state of fitness.

Our results indicated that patients who had more disease-related worried were more likely to have higher levels of overall supportive care needs, psychological needs, patient care and supportive needs, and sexuality needs. These findings may be explained as being related to this time period of the interval between patients going home after outpatient chemotherapy until their next treatment, as well as the fact that concerns were related to treatment-related side effects, cancer progression, and treatment response. During this interval period, support related to worrying might not have been sufficiently tailored to meet patients’ expectations. In the clinical setting, we should individualize supportive guidance in order to address patient-centered expectations and help patients understand the treatment program and assist patients in dealing with treatment side-effects.

The study results indicated that body image was one of the predictors of physical and daily living needs. More than four-fifths of our patients were of advanced stage and receiving multimodal treatments. These findings agree with those of previous studies of postoperative and chemotherapy patients [31,34]. These findings may reflect that an aspect of UGI organ resection resulted in patients who perceived alternation of body image due to changed appearance of their skin, disruption of soft tissue, or organ disfunction. Moreover, after chemotherapy, patients may experience dissatisfaction with their appearance due to side effects such as hair loss, skin and nail color change, or weight loss. Diet modification that addresses weight loss, as well as careful choice of clothing worn, may contribute towards patients developing a more positive body image.

The study also found that younger patients perceived greater psychological needs than older patients, consistent with an earlier study [15]. Our own clinical observations are that younger patients had heavier family and economic responsibilities, while older patients were moving towards retirement. Healthcare providers should therefore actively evaluate patients’ concerns and encourage patients to express their feelings.

Results of the present study revealed that patients with lower physical performance had higher overall supportive care needs, as well as physical and daily living needs, than those with better physical performance, which is consistent with previous research results [35]. These results indicate that both unmet needs and physical self-care vary with level of physical functioning and self-care ability. Home physical activities can help to address functional decline and assist patients in meeting their overall supportive care needs and physical and daily living needs.

This study has several limitations. First, the present study examined UGI cancer patients who received at least two cycles of treatment within the last three months; however, GI symptom distress, and supportive care needs following chemotherapy cycles, is a dynamic process that changes over time. Longitudinal, or long-term follow-up studies, are needed to identify characteristics and changes in GI symptom distress and supportive care needs. Second, we did not consider individual independence or personality as factors in unmet needs. Thus, future studies should include individual independence and personality characteristics in their analysis. Third, family support, and available resources in the home may impact unmet needs, but we did not consider these factors. Comparative studies are needed to determine the effect of supportive systems on unmet needs among patients receiving outpatient chemotherapy. Fourth, since the sample size was small, our results may not be generalizable to other settings. Future studies with larger sample sizes are needed to further examine the predictors of this test. Finally, the instrument used to assess symptom distress, the EORTC-QLQ-GINET21, may not have fully captured the extent of all parameters with the existing scales. Future studies are needed to explore psychometric properties among different sites of GI cancer.

## 5. Conclusions

In conclusion, the SCNS-ST9 screened the priorities of unmet supportive care needs as follows: the top unmet needs were “fears about the cancer spreading”, “uncertainty about the future”, and “being informed about things you can do to help yourself to get well”. Disease-related worries, treatment-related symptoms, and physical performance were found to be associated with supportive care needs among UGI cancer patients receiving chemotherapy. The evaluative scale utilized is relatively brief and easily used in clinical settings. Patient satisfaction and service delivery will be improved if all patients are screened for unmet needs.

Our study documents the large degree of unmet needs, as well as significant factors regarding supportive care needs, in UGI cancer patients who received outpatient treatments. In a clinical setting, we were able to use a relatively simple tool to quickly screen patients’ unmet needs and promptly manage problems in order to meet their needs.

## Figures and Tables

**Table 1:** Demographic and clinical characteristics of patients (N = 100).

Variable	Number (%)	Mean (Median)	IQR
Age	-	60 (62)	55–67
Gender	-	-	-
Male	58 (58)	-	-
Female	42 (42)	-	-
Employment after diagnosis	-	-	-
Unemployed	44 (44)	-	-
Quit job after diagnosis	46 (46)	-	-
Return to work after discharge	10 (10)	-	-
Marital status	-	-	-
Unmarried	5 (5)	-	-
Married	95 (95)	-	-
Educational level	-	-	-
None	7 (7)	-	-
Elementary	30 (30)	-	-
Junior high	22 (22)	-	-
Senior high	28 (28)	-	-
College and above	13 (13)	-	-
Religion	-	-	-
None	26 (26)	-	-
Buddhism/Taoism	66 (66)	-	-
Christianity	6 (6)	-	-
Other	2 (2)	-	-
Smoking	-	-	-
Nil/ex-smoker	85 (85)	-	-
Current smoker	15 (15)	-	-
Drinking	-	-	-
Nil/ex-drinker	99 (99)	-	-
Current drinker	1 (1)	-	-
Diagnosis	-	-	-
Gastric cancer	43 (43)	-	-
Pancreas cancer	25 (25)	-	-
Cholangiocarcinoma	29 (29)	-	-
Other	3 (3)	-	-
Cancer stage	-	-	-
I	1 (1)	-	-
II	16 (16)	-	-
III	30 (30)	-	-
IV	53 (53)	-	-
Recurrence	-	-	-
No	94 (94)	-	-
Yes	8 (8)	-	-
Medical treatment	-	-	-
Surgery + CT	53 (53)	-	-
Surgery + CCRT	1 (1)	-	-
CT	45 (45)	-	-
CCRT	1 (1)	-	-
Performance status (KPS level)	-	-	-
70 to 80	3 (3)	-	-
80 to 90	23 (23)	-	-
90 to 100	74 (74)	-	-
Time since cancer diagnosis (week)	-	15.57 (12)	8–16

IQR, interquartile range.

**Table 2:** Scores for GI symptom distress, social support, and supportive care needs (N = 100).

Variable	Mean	Median	IQR
GI symptom distress (EORTC-QLQ GINET21)	-	-	-
- Muscle/bone pain (MBP)	12.33	0	0–33.33
- Body image (BI)	13.33	0	0–33.33
- Information/communication function (INF)	50.02	66.67	33.33–66.67
- Sexual function (SX)	11.33	0	0–25
- Endocrine symptom (ED)	5.22	0	0–11.11
- GI symptom (GI)	12.88	13.33	0–20
- Treatment-related symptom (TR)	16.27	11.11	0–27.78
- Social functioning (SF)	16.11	11.11	11.11–22.22
- Disease-related worries (DRW)	23.68	22.22	11.11–33.33
Social support (SSS)	90.87	96.05	87.17–100
- Emotional/informational support	79.83	86.11	75.69–88.89
- Tangible support	40.97	44.44	38.89–44.44
- Affectionate support	92.83	100	91.67–100
- Positive social interaction	90.25	100	83.33–100
Supportive care needs (overall)(SCNS-ST9)	34.25	25	19.44–35.42
- Psychological needs	34.13	25	25–50
- Health information needs	34.75	25	25–34.38
- Physical and daily living needs	27.13	25	25–50
- Patient care and supportive needs	13.00	25	25–25
- Sexuality	29.72	0	0–25

EORTC-QLQ GINET21, European Organization for Research and Treatment of Cancer—Gastrointestinal-Related Neuroendocrine Tumors; MBP, muscle and/or bone pain; BI, body image; INF, information/communication function; SX, sexual functioning; ED, endocrine symptoms, GI, GI symptoms; TR, treatment-related symptoms; SF, social functioning; DRW, disease-related worries. SSS, Social Support Scale. SCNS-ST9, Supportive Care Needs Survey-Screening Tool; IQR, interquartile range.

**Table 3:** Prevalence of unmet supportive care needs (N = 100).

Rank	Unmet Supportive Care Needs ^a^	Domain	%
1	Fears about the cancer spreading	Psychological needs	15.0
2	Uncertainty about the future	Psychological needs	14.0
3	Being informed about things you can do to help yourself to get well	Health information needs	11.0
4	Not being able to do the things you used to do	Physical and daily living needs	9.0
5	Being informed about your test results as soon as feasible	Health information needs	8.0
6	Lack of energy and tiredness	Physical and daily living needs	8.0
7	Changes in your sexual relationships	Sexuality	3.0
8	Hospital staff acknowledging, and showing sensitivity to, your feelings and emotional needs	Patient care and supportive needs	2.0
9	Reassurance by medical staff that the way you feel is normal	Patient care and supportive needs	2.0

^a^ Unmet needs are defined as the score of the item 4 (moderate need) to 5 (high need).

**Table 4:** Factors significantly associated with overall supportive care needs and five aspects of supportive care needs in terms of multiple regression analysis (N = 100).

Domains of Supportive Care Needs	Predictive Variable	R^2^	Beta	95% CI	
				Lower	Upper
Overall supportive care needs (SCNS-ST9)	Disease-related worries (DRW)	0.480	0.428	0.173	0.408
	Treatment-related symptom (TR)	-	0.331	0.122	0.371
	Physical performance	-	−0.188	−0.920	−0.080
	Constant	-	-	24.541	98.861
Psychological needs (SCNS-ST9)	Disease-related worries (DRW)	0.539	0.377	0.209	0.636
	Treatment-related symptom (TR)	-	0.251	0.115	0.502
	Endocrine symptom (ED)	-	−0.185	−0.908	−0.112
	Social functioning (SF)	-	0.234	0.088	0.725
	Age	-	−0.149	−0.614	−0.014
	Constant	-	-	14.741	52.601
Health information needs (SCNS-ST9)	Social functioning (SF)	0.332	0.362	0.282	0.861
	Treatment-related symptom	-	0.183	−0.003	0.411
	GI symptom (GI)	-	0.191	0.013	0.589
	Marital status	-	0.175	0.090	19.743
	Constant	-	-	0.0321	16.207
Physical and daily living needs (SCNS-ST9)	Body image (BI)	0.390	0.255	0.070	0.399
	Social functioning (SF)	-	0.218	0.051	0.508
	Physical performance	-	−0.303	−1.562	−0.392
	Treatment-related symptom (TR)	-	0.183	0.007	0.325
	Constant	-	-	56.860	161.210
Patient care and supportive needs (SCNS-ST9)	Disease-related worries (DRW)	0.204	0.313	0.062	0.277
	Sexual function (SX)	-	0.233	0.019	0.210
	Constant	-	-	18.260	24.190
Sexuality needs (SCNS-ST9)	Disease-related worries (DR)	0.338	0.204	0.009	0.387
	Sexual function (SX)	-	0.272	0.079	0.413
	Cancer stage	-	−0.203	−19.919	−1.623
	Treatment-related symptom (TR)	-	0.216	0.030	0.430
	Constant	-	-	0.210	19.963

SCNS-ST9, Supportive Care Needs Survey-Screening Tool. Input independent variable: covariates included: age (continuous score); marital status (no vs. yes); religion (no vs. yes), cancer stage (early vs. advanced); physical performance (continuous score); muscle bone pain (MBP) (continuous score); body image (BI) (continuous score); information/communication function (INF) (continuous score); sexual function (SX) (continuous score); endocrine symptoms (ED) (continuous score); GI symptoms (GI) (continuous score); treatment-related symptoms (TR) (continuous score); social functioning (SF) (continuous score); disease-related worries (DRW) (continuous score); and social support (continuous score).

## Data Availability

The data that support the findings of this study are available from the corresponding author. Restrictions apply to the availability of these data, which were used under license for this study. Data are available from the authors with the permission of the Chang Gung Memorial Hospital Research Program (CMRP).
